# OsCRLK2, a Receptor-Like Kinase Identified by QTL Analysis, is Involved in the Regulation of Rice Quality

**DOI:** 10.1186/s12284-024-00702-2

**Published:** 2024-04-08

**Authors:** Ying Chen, Hanfeng Shi, Guili Yang, Xueyu Liang, Xiaolian Lin, Siping Tan, Tao Guo, Hui Wang

**Affiliations:** National Engineering Research Center of Plant Aerospace-mutation Breeding, South China Agricultural University, 510642 Guangzhou, China

**Keywords:** Rice quality, QTL, Candidate gene, Receptor-like protein kinases

## Abstract

The quality of rice (*Oryza sativa* L) is determined by a combination of appearance, flavor, aroma, texture, storage characteristics, and nutritional composition. Rice quality directly influences acceptance by consumers and commercial value. The genetic mechanism underlying rice quality is highly complex, and is influenced by genotype, environment, and chemical factors such as starch type, protein content, and amino acid composition. Minor variations in these chemical components may lead to substantial differences in rice quality. Among these components, starch is the most crucial and influential factor in determining rice quality. In this study, quantitative trait loci (QTLs) associated with eight physicochemical properties related to the rapid viscosity analysis (RVA) profile were identified using a high-density sequence map constructed using recombinant inbred lines (RILs). Fifty-nine QTLs were identified across three environments, among which *qGT6.4* was a novel locus co-located across all three environments. By integrating RNA-seq data, we identified the differentially expressed candidate gene *OsCRLK2* within the *qGT6.4* interval. *osclrk2* mutants exhibited decreased gelatinization temperature (GT), apparent amylose content (AAC) and viscosity, and increased chalkiness. Furthermore, *osclrk2* mutants exhibited downregulated expression of the majority of starch biosynthesis-related genes compared to wild type (WT) plants. In summary, *OsCRLK2*, which encodes a receptor-like protein kinase, appears to consistently influence rice quality across different environments. This discovery provides a new genetic resource for use in the molecular breeding of rice cultivars with improved quality.

**Supplementary Information:**

The online version contains supplementary material available at 10.1186/s12284-024-00702-2.

## Background

Rice (*Oryza sativa* L.) is one of the most important grain crops, serving as the primary source of calories for over half of the world’s population (Zhou et al. 2016). Beginning in the 1950s, rice breeding has experienced three major technological revolutions, leading to a significant increase in grain yield (Sasaki et al. 2002). Now that yields are high enough to meet the needs of the average consumer, consumers are beginning to focus their attention on rice quality (Buenafe et al. 2021). However, genetic complexity and challenges associated with the accurate assessment of grain quality have impeded progress in the development of improved rice cultivars (Rebeira et al. 2014). Utilizing molecular techniques to discover genes and regulatory networks associated with grain quality will expedite the optimization of rice cultivars with superior quality characteristics (Yun et al. 2016).

Starch content and composition heavily influence rice grain quality. Starch can be broadly categorized into two structural components, amylose and amylopectin, and their compositions and structures directly impact the physical properties of the starch and the quality of the rice grain (Hori and Sun 2022). Starch composition and structure are most commonly studied using methods such as iodine colorimetry, particle size analysis, X-ray diffraction (XRD), ion chromatography, and nuclear magnetic resonance (NMR) spectroscopy, among other techniques. Additionally, the physical (i.e., viscosity) and thermodynamic properties of starch can be measured directly using methods such as rapid viscosity analysis (RVA), differential scanning calorimetry (DSC), and dynamic rheometry, among others. These analyses allow researchers to characterize the properties of starch in order to better understand their relationship with rice quality.

Recent years, in-depth studies have been conducted to investigate the composition, structure, and physical properties of starch, utilizing diverse population materials. Successful identification and cloning of numerous loci and genes influencing the quality of rice have been achieved as a result. For example, Su et al. (Su et al. 2011) used a double haploid (DH) population to map a locus (*qGC-6*) impacting gel consistency (GC) and a chromosome segment substitution line (CSSL) to identify a candidate gene (*Wx*) controlling grain amylose content as well as GC. Gao et al. (Gao et al. 2003, 2011) used map-based cloning to identify the gene (*ALK*) responsible for establishing the gelatinization temperature (GT). Notably, their research suggested that base substitutions within the *ALK* coding region may induce changes in the amylopectin crystal structure, thus affecting the GT (evaluated by alkali digestion) (Gao et al. 2003, 2011). The *Flo5* gene also influences the structure of amylopectin, the content of amylose, and the physicochemical properties of starch in rice grains (Fujita et al. 2007). In addition, 19 quantitative trait loci (QTLs) associated with the RVA profile were identified using recombinant inbred lines (RILs) (Hsu et al. 2014). Among these, five QTLs located on chromosome 6 (Chr6) were found to be related to trough viscosity (TV), breakdown value (BDV), final viscosity (FV), setback value (SBV), and peak time (PKT), located near the gene *Wx* and their contributions to the phenotypic variance are all above 55% (Hsu et al. 2014). Finally, research using a multi-parent advanced generation intercross (MAGIC) population resulted in the identification of 13 QTLs related to the RVA profile (Ponce et al. 2018). Among these, *qPKT6.1*, *qPKV6*, *qBDV6.1*, *qTV6.1*, *qCPV6*, and *qSBV6* were identified at or near the gene *Wx* (Ponce et al. 2018).

In this study, we used a high-density genetic map to identify QTLs associated with RVA parameters in a RIL population across three different environments. A total of 59 RVA-associated QTLs were detected, including 10 genes previously reported to be associated with starch biosynthesis and spikelet development, among other related traits: *MFS2* (Deyong et al. 2020), *OsMADS34* (Kobayashi et al. 2010), *SPDT* (Yamaji et al. 2017), *OsGWD1* (Wang et al. 2021), *OsMFT1* (Song. et al. 2018), *OsFAD2-1* (Tiwari et al. 2016), *OsNF-YA4* (Lee et al. 2015), *ALK* (Gao et al. 2003), *YPD1* (Chen et al. 2020), and *TGW6* (Ishimaru et al. 2013). By integrating the transcriptomic sequencing results, we identified a candidate gene (*OsCRLK2*) in the *qGT6.4* region which consistently influenced rice quality under diverse environmental conditions. Specifically, *OsCRLK2* encodes a receptor-like protein kinase and *osclrk2* mutants exhibited reduced GT and apparent amylose content (AAC). Analysis of *OsCRLK2* using the Rice Functional Genomics and Breeding (3K RFGB) database and the Rice Haplotype Reference Database (RHRD) revealed pronounced genetic differentiation between *indica* and *japonica* rice varieties. In addition, different haplotypes may impact both yield and grain quality. This research provides novel insights into the molecular genetics underpinning rice quality.

## Materials and Methods

### Plant Materials

A linkage mapping population consisting of 192 RILs was derived from a cross between the *indica* cultivar ‘PYZX’ and the *japonica* cultivar ‘P02428’. Both parental cultivars and the RILs were grown at the Tianhe District Breeding Experimental Base of the National Plant Aerospace Breeding Engineering and Technology Research Center of South China Agricultural University in February 2017 (E1), July 2017 (E2), and March 2021 (E3). The experimental field was consistently managed in accordance with local cultivation practices.

### Phenotypic Analyses of Rapid Viscosity Analysis-Associated Traits

Phenotypic analyses of RVA-associated traits were conducted in rice grown in three environments. Briefly, mature rice grains were harvested, air dried, and stored at room temperature for three months prior to grinding (100 mesh) into whole rice flour. The AAC was measured using standard iodine colorimetry (Bao et al. 2006b). Peak viscosity (PKV), TV, FV, and PKT, as well as and the derivatives BDV (PKV-TV), SBV (FV-PKV), and consistency viscosity (CSV, FV-TV), were quantified using RVA. The data were sorted with Thermal Cycle for Windows (TCW) (Bao et al. 2006b). The GT was estimated using Bao’s formula (PT_m_ = (45/3.8)×(T_1_-1) + 50) (Bao 2008) because Bao’s study reported a highly significant positive correlation (*r* = 0.97) between the manually calculated PT_m_ and the peak temperature (Tp) determined by DSC. Viscosity was expressed in units of centipoise (cP).

### Mapping of Quantitative Trait Loci

A high-density genetic map containing 2,711 recombination bin markers was constructed using 85,743 high-quality single nucleotide polymorphisms (SNPs) obtained from Novogene Bioinformatics Technology Co., Ltd. (Beijing, China) (Chen et al. 2016). QTLs were identified based on interval mapping (IM) with ICIMapping (Ver. 4.2) (http://www.isbreeding.net), with the following parameters: scanning step = 1 cM, and limit of detection (LOD) = 2.5. Genes within the mapping intervals were annotated using the Ensembl Plants (http://plants.ensembl.org/index.html) and Rice Genome Annotation Project (http://rice.uga.edu/index.shtml) databases. In this study, QTL nomenclature followed that of the Committee on Gene Symbolization, Nomenclature and Linkage (CGSNL, Rice Genetics Cooperative) and McCouch (McCouch 2008).

### Quantitative Real-Time Polymerase Chain Reaction Analysis

Total RNA was extracted using an EASY Spin Plus Complex Plant RNA Kit (Aidlab Biotechnologies Co., Ltd., Beijing, China), according to the manufacturer’s instructions. Reverse transcription was performed using a GoScript Reverse Transcription System (Promega Biology Co., Ltd., WI, USA), according to the manufacturer’s instructions. Quantitative real-time polymerase chain reactions (qRT-PCR) were carried out using the AceQ qPCR SYBR Green Master Mix (Vazyme, Nanjing, China) and an ABI Step One Plus Real-Time PCR System (Applied Biosystems, MA, USA), according to the manufacturer’s instructions. All experiments were conducted with three biological replicates. Data are shown as means ± standard deviation (SD). The relative expression level of candidate genes was quantified using the 2^−∆∆CT^ method (Livak and Schmittgen 2001). All primers are listed in Table S1.

### Vector Construction and Production of Transgenic Plants

Clustered regularly interspaced short palindromic repeats CRISPR/Cas9 vectors for *OsCRLK2* were constructed as previously described (Xie et al. 2015, 2017 ). The pRGEB32-*OsCRLK2* plasmid was transferred into *Agrobacterium tumefaciens* strain ‘EHA105’ for infection of Nipponbare (Nip) callus. Transgenic plants were screened using the sequence of hygromycin and target sites. All transgenic materials were propagated to the T_3_ or T_4_ generation for phenotypic analyses. CRISPR-based genome editing (GE) (http://skl.scau.edu.cn/) was used to design target subgenomic mRNAs (sgRNAs) and predict potential off-target sites. All primers are listed in Table S1.

### Subcellular Localization Assays

The *OsCRLK2* coding sequence (CDS) was cloned into pAN580 to generate the p35S: *OsCRLK2*-GFP vector. Rice protoplasts were isolated as previously described (Yang et al. 2014). After culturing the protoplasts for 12 h in darkness at 26°C, they were observed under an LSM800 laser scanning confocal microscope (Carl Zeiss, Oberkochen, Germany) to detect fluorescence. All primers are listed in Table S1.

### Observation of Starch Granule Structure

Starch granules were coated with gold-palladium using an EM ACE600 vacuum sputter coater (Leica, Wetzlar, Germany) and observed at 4500× resolution with an EVO MA 15 scanning electron microscope (SEM) (Carl Zeiss, Oberkochen, Germany) operating at an accelerating voltage of 10 kV.

### Evaluation of Chalkiness, AAC, Amylose and Amylopectin Content, and Sucrose Content

The degree of grain chalkiness is defined as the percentage of the total chalky area of chalky rice grains relative to the total area of head rice in the sample. The degree of chalkiness was evaluated using a WSeen measuring system (SC-E, Wanshen Testing Technology Co., Ltd., Hangzhou, China), as well as Chalkiness1.0 (Hunan Provincial Key Laboratory of Phytohormones), for assessing rice grain quality. The AAC was measured using iodine colorimetry (Li et al. 2020). The precise contents of amylose and amylopectin were quantified using a commercial Amylose/Amylopectin/Total Starch Content kit (Aidisheng Biotechnology Co., Ltd., Jiangsu, China), according to the manufacturer’s instructions. This assay kit exploits the specific binding of concanavalin A to amylopectin but not to amylose, facilitating the separation of amylose and amylopectin. By utilizing total starch content as the denominator, direct quantification of amylose and amylopectin content was achieved. Sucrose content was quantified using the anthrone colorimetric method (Aidisheng Biotechnology Co., Ltd., Jiangsu, China), according to the manufacturer’s instructions. AAC, actual amylose and amylopectin content, as well as sucrose content, were all determined using milled rice.

## Results

### Phenotypes of Parental Cultivars and Recombinant Inbred Lines

A RIL population was derived by single seed descent from a cross between the *indica* cultivar ‘PYZX’ and the *japonica* cultivar ‘P02428’. These parental cultivars were chosen because they exhibited significant variation in multiple rice quality traits. We assessed the AAC and eight RVA-associated physicochemical properties in both the parental cultivars and RILs.

**Fig. 1:**
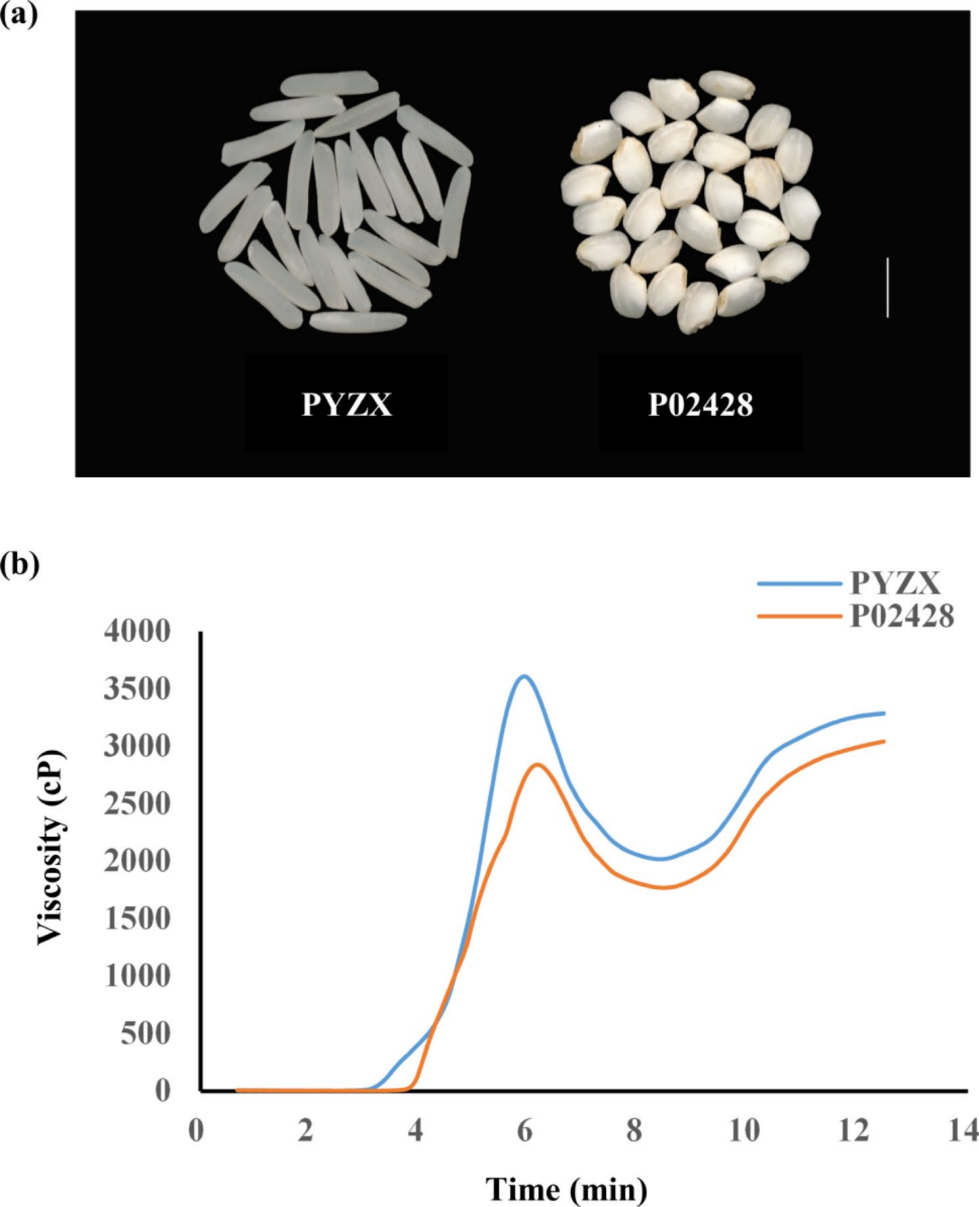
Phenotypic differences between ‘PYZX’ and ‘P02428’. (**a**) Milled grains (Bar = 5 mm). (**b**) RVA curve of the parent

Both ‘PYZX’ and ‘P02428’ exhibited similar AACs (13.9% ± 1% and 11.8% ± 1.7%, respectively) in Guangzhou from 2017 to 2021. However, considerable variation in chalkiness was observed between the two parental cultivars. Specifically, the point at which the pasting viscosity begins to increase occurred earlier in ‘PYZX’ than in ‘P02428’ (Fig. 1).

We statistically analyzed the PKV, TV, FV, PKT, and GT of the RILs and discovered that the two parental cultivars exhibited different viscosity characteristics. Notably, the PKV, TV, and FV of the RILs had a larger coefficient of variation during the early season compared to the late season, indicating that most of the RVA parameters were influenced by environmental factors (Table S2).

In the RILs, the eight RVA-associated physicochemical properties exhibited continuous variation, transgressive segregation, and a normal distribution across the three environments. These results indicate that these indices are quantitative traits controlled by multiple factors. Notably, GT exhibited a bimodal distribution in the RIL population (Fig.S1), suggesting that GT is primarily controlled by a major locus as well as some multitude of minor loci.

According to our Pearson’s correlation analysis, we found significant positive correlations between AAC and TV, AAC and FV, AAC and SBV, as well as AAC and CSV across all three environments, while also exhibiting a significant negative correlation between AAC and GT. Additionally, within the RIL population, PKV showed significant positive correlations with TV and FV, while TV exhibited a significant positive correlation with FV (Table S3).

### Quantitative Train Loci Mapping Based on the High-Density Genetic Map

**Fig. 2:**
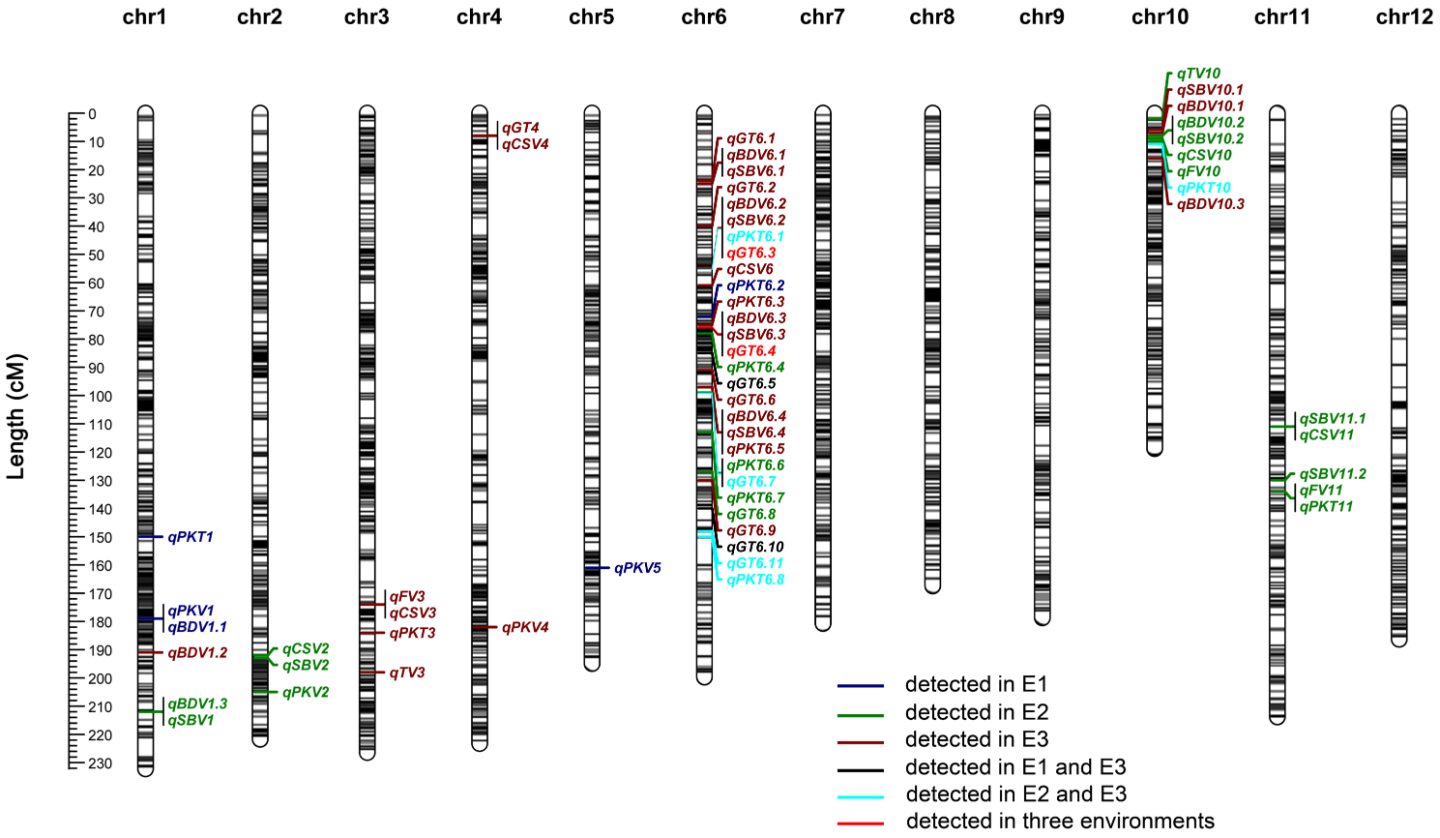
Distribution of detected QTLs associated with eight physicochemical properties in the high-density genetic map. Black lines represent the positions of bin markers in each linkage group. Dark blue, green, and brown shapes represent E1, E2, and E3, respectively. Black lines indicate QTLs detected in E1 and E3, light blue lines indicate QTLs detected in E2 and E3, and red lines indicate QTLs detected in E1, E2, and E3

A total of 59 rice quality-associated QTLs were identified across eight chromosomes in three environments (Fig. 2 and Table S4). Specifically, four QTLs were associated with PKV, two QTLs were associated with TV, and three QTLs were associated with FV. Twelve QTLs controlling PKT were identified, among which *qPKT6.1*, *qPKT6.8*, and *qPKT10* were all located simultaneously in E2 and E3. Twelve QTLs controlling GT were located on Chr6, with the exception of *qGT4*. Among them, *qGT6.3* and *qGT6.4* were repeatedly detected in all three environments (LOD > 7). *qGT6.3* and *qGT6.4* were located from 6.55 to 6.65 Mb and 9.95 to 10.05 Mb, respectively, on Chr6. On average, *qGT6.3* was found to explain 16.2491% of the phenotypic variation, while *qGT6.4* was found to explain 9.1492% of the phenotypic variation. *qGT6.5* and *qGT6.10* were detected in both E1 and E3, and *qGT6.7* and *qGT6.11* were detected in both E2 and E3. However, *qGT6.5*, *qGT6.10*, *qGT6.7*, and *qGT6.11* individually explained less than 7% of the phenotypic variation.

### Identification of Candidate Genes

Since *qGT6.3* and *qGT6.4* were repeatedly detected across all three environments, we speculated that they may contain genes which can stabilize inheritance and affect GT.

As mentioned, GT exhibited a bimodal distribution in the RIL population. *qGT6.3* was found to explain > 15% of the phenotypic variation in both environments, suggesting that a major gene controlling GT may be located in this interval. Using the 3K RFGB (https://www.rmbreeding.cm/index.php), we found a cloned gene (*ALK*) which regulates GT and is located in the region 100 Kb downstream of the 6.55–6.65 Mb interval. We compared the *ALK* sequences of ‘PYZX’ and ‘P02428’ in combination with the re-sequencing data (from 3 K RFGB). Two SNPs were identified in exon 8 (Ex8), at positions Ex8-864/865 bp (G/T and C/T) between the parental cultivars. ‘P02428’ belongs to the *ALK*^*c*^ (G-GC) haplotype (high GT), while ‘PYZX’ belongs to the *ALK*^*b*^ (G-TT) haplotype (low GT).

**Fig. 3:**
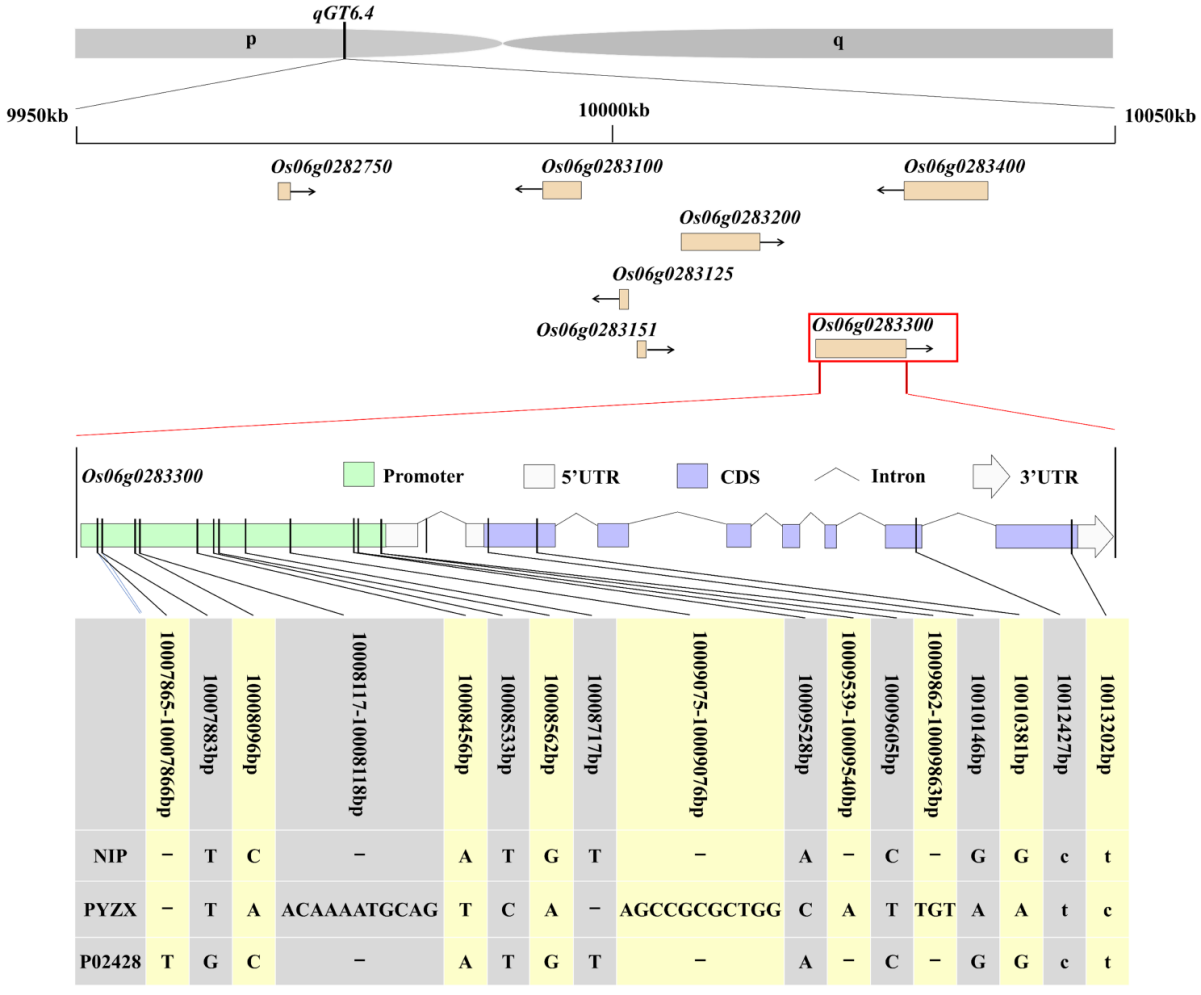
Identification of candidate genes and SNPs among parental cultivars. Lowercase letters indicate synonymous mutations and uppercase letters indicate nonsynonymous mutations

The *qGT6.4* locus has not yet been reported, and is therefore considered a novel QTL affecting GT. According to the Rice Genome Annotation Project (https://rapdb.dna.affrc.go.jp/download/irgsp1.htm), seven genes were annotated in the *qGT6.4* region (Fig. 3). Using the parental RNA-seq data generated during the filling period 20 days after flowering (DAF20), we screened one DEG: *Os06g0283300*. Consequently, it was hypothesized that *Os06g0283300* may be a candidate gene for *qGT6.4*. *Os06g0283300* exhibited high sequence homology with *Arabidopsis AtCRLK2* (*AT5G15730*), and was therefore designated *OsCRLK2*.

Based on sequence alignment in both ‘PYZX’ and ‘P02428’, four SNPs were identified in the CDS and ten SNPs along with three indels were identified in the promoter of *OsCRLK2* (Fig. 3). These sequence variations, including SNPs, deletions, and insertions, may impact both protein function and gene expression.

### The Highly-Conserved Gene *OsCRLK2* Encodes a Receptor-like Kinase

Bioinformatics analysis revealed that *OsCRLK2* encodes a 434 amino-acid protein. The HMMER web server (https://www.ebi.ac.uk/Tools/hmmer/) predicted that the OsCRLK2 protein contains a transmembrane domain and an intracellular juxtamembrane domain (Fig.S2). Combined with the annotation analysis, the protein is likely to be a receptor-like kinase (RLK).

**Fig. 4:**
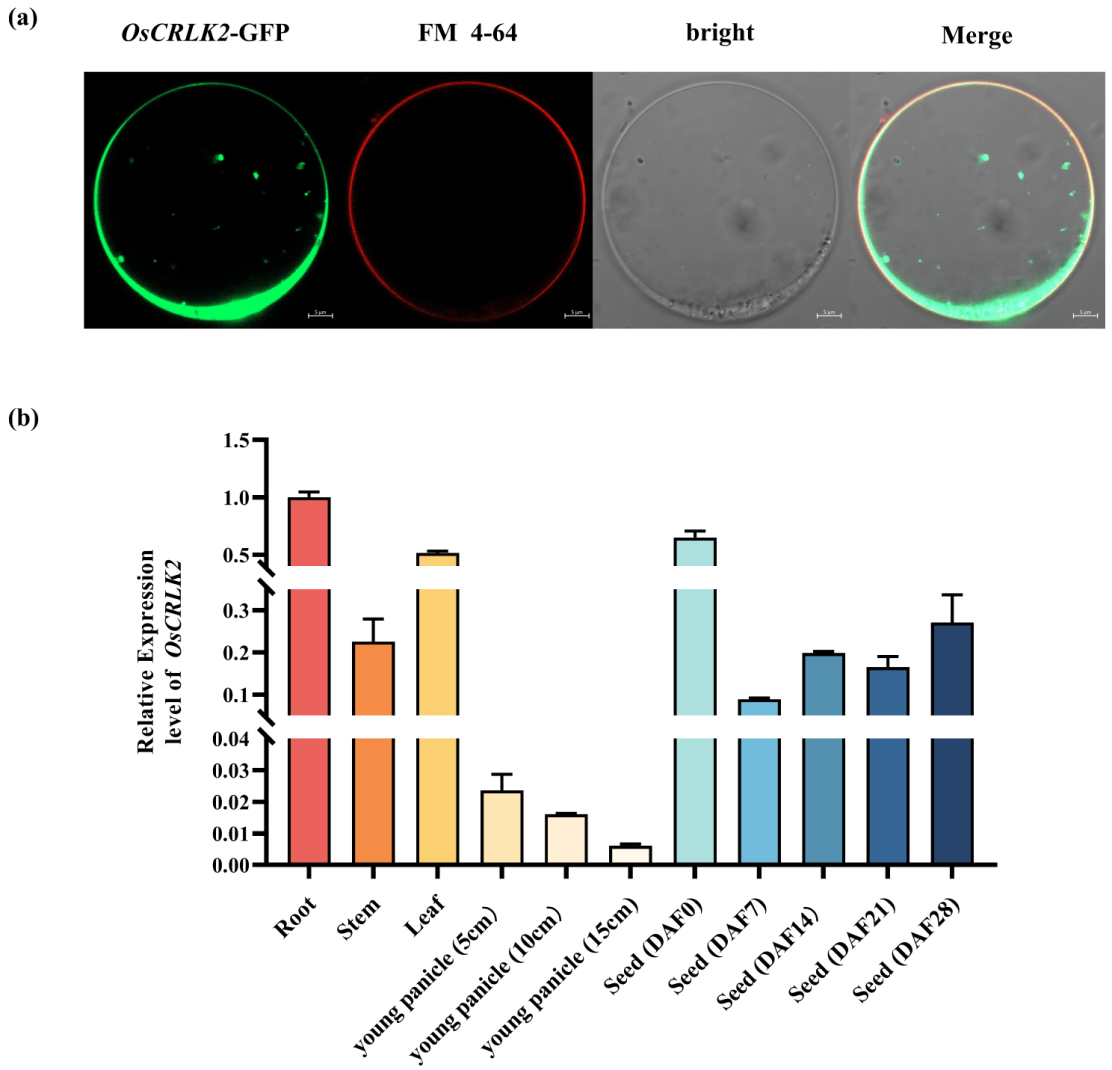
Expression pattern of *OsCRLK2*. (**a**) Subcellular localization of OsCRLK2-GFP in rice protoplasts (Bar = 5 μm). (**b**) Relative expression levels of *OsCRLK2* in different tissues and developing endosperm at 0–28 DAF. *OsActin* was used as an internal control. Values are shown as means ± SD (*n* = 3)

We further performed an amino acid sequence alignment and constructed a phylogenetic tree for OsCRLK2. Specifically, the multiple sequence alignment showed that OsCRLK2 exhibited high sequence conservation, with multiple highly homologous proteins identified in different species (Fig.S3). The ubiquity of this protein and high similarity observed between OsCRLK2 homologs implies that this protein may perform a fundamentally important biological function. Transient expression in rice protoplasts showed that OsCRLK2 was primarily localized to the membranes and cytoplasm (Fig. 4a). Moreover, qRT‒PCR analysis revealed that *OsCRLK2* was expressed in multiple tissues, with the highest levels observed in roots, leaves, and seeds (DAF0) (Fig. 4b).

### *OsCRLK2* Regulates Rice Quality

**Fig. 5:**
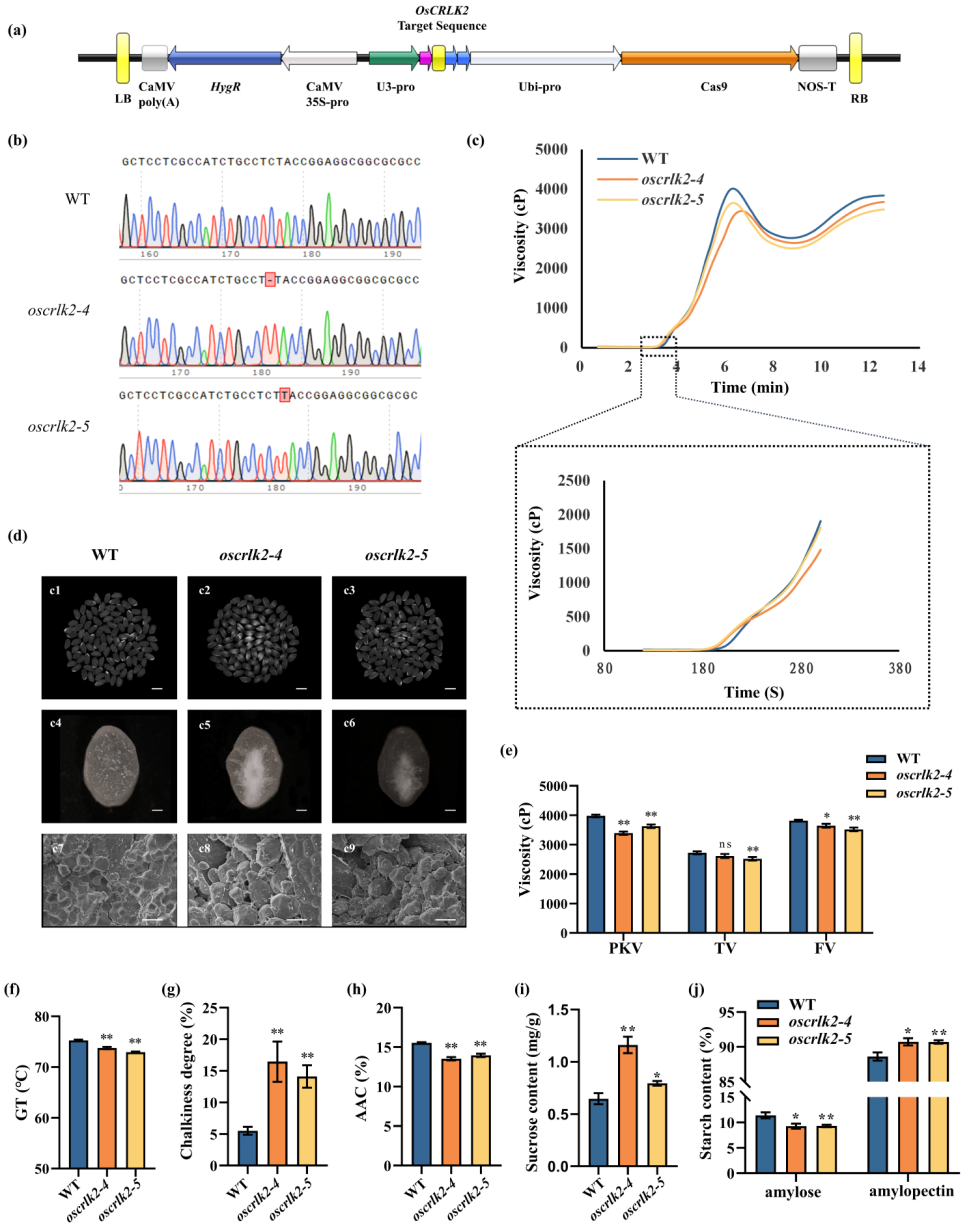
Phenotypic characterization of *oscrlk2*. (**a**, **b**) Creation and detection of *oscrlk2.* (**c**) RVA curve of WT and *oscrlk2* mutants. (**d**) Milled rice morphology of c1-c3 (Bar = 5 mm). Grain appearance was evaluated using the WSeen analysis system. The white milled rice grains in the middle are the chalky grains. Transverse sections of mature seeds from c4-c6 (Bar = 0.5 mm). SEM images of transverse sections of mature seeds from c7-c9 (Bar = 10 μm). (**e**–**j**) Viscosity (cP)\, GT (°C), chalkiness degree (%), AAC (%), sucrose contents (mg/g), amylose and amylopectin contents (%) in mature endosperm of WT and *oscrlk2* mutants. Values are shown as means ± SD (*n* = 3). **P < 0.01 and *P < 0.05 (Student’s *t* test)

To characterize the role of *OsCRLK2* in the regulation of rice quality, we used CRISPR/Cas9 to generate *OsCRLK2* mutants in the *japonica* Nipponbare background. Specifically, we generated two *oscrlk2* alleles introducing premature stop codons: *oscrlk2-4* with a 1-bp deletion and *oscrlk2-5* with a 1-bp insertion (Fig. 5a, b).

**Fig. 6:**
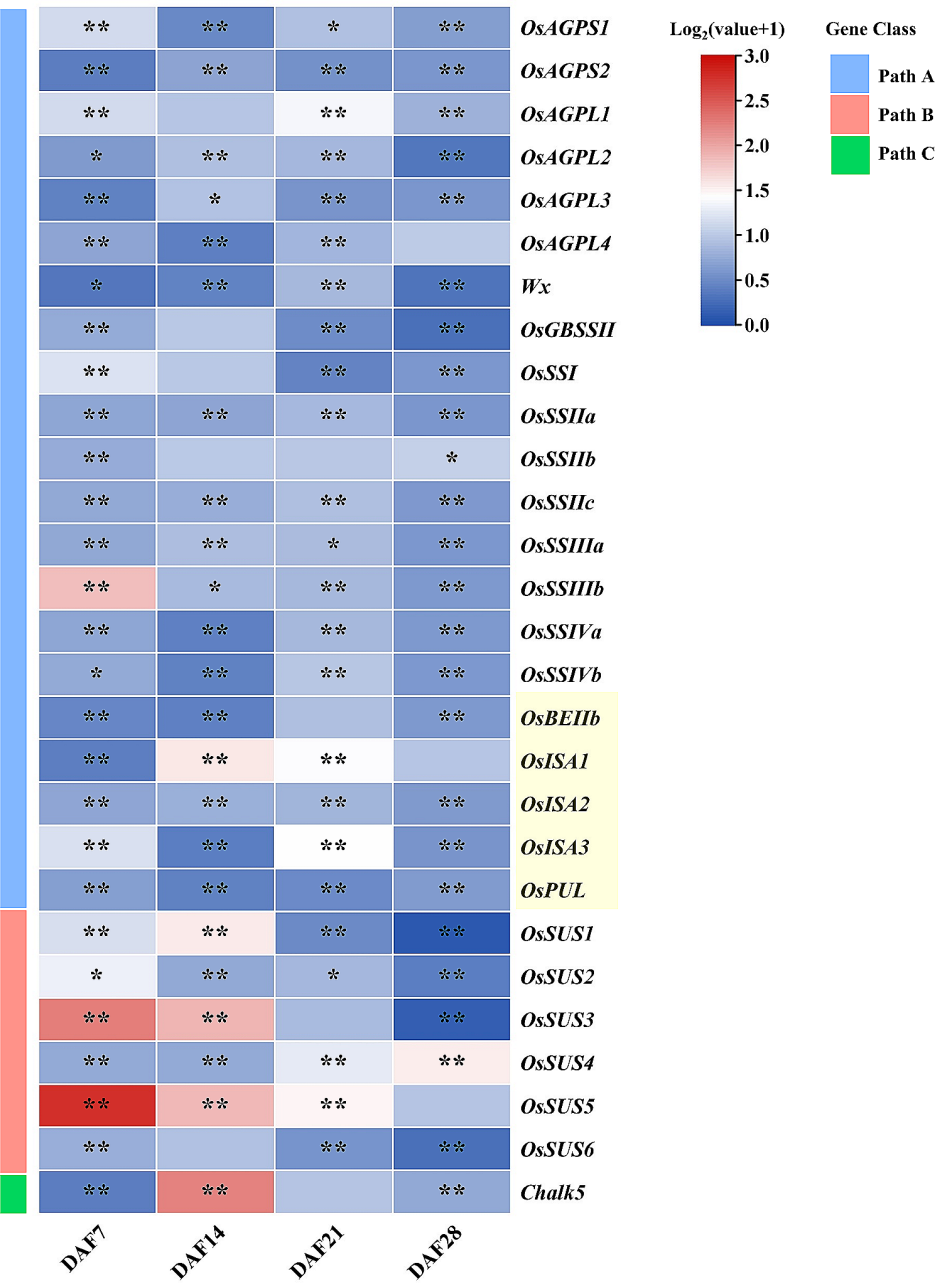
The relative expression levels of starch synthesis-related and sucrose synthase genes in developing endosperm at 7-28 DAF of WT and *oscrlk2*. Part A encompasses genes related to starch synthesis, with those highlighted in yellow indicating branching and debranching enzymes involved in amylopectin synthesis. Part B consists of the sucrose synthase genes, while Part C consists of key genes regulating chalkiness. *OsActin* was used as an internal control. Values are shown as means ± SD (*n* = 3). **P < 0.01 and *P < 0.05 (Student’s *t* test).

Phenotypic characterization revealed that the point at which the pasting viscosity begins to increase occurred earlier in the mutants than in the wild type (WT) plants, resulting in lower GT in *oscrlk2* (Fig. 5c, f). In addition, compared to WT plants, the mutants exhibited greater chalkiness; non-compact starch granules; and lower AAC, PKV, and FV (Fig. 5d, e, g, h). Moreover, the contents of amylopectin and sucrose were higher in the mutants than in the WT plants (Fig. 5i, j). According to qRT‒PCR analysis, the majority of starch biosynthesis-related genes were downregulated, while some sucrose synthase genes were upregulated, during the grain filling period (DAF7-DAF28) in *oscrlk2* compared to the WT. Additionally, some genes encoding soluble starch synthase, branching enzymes and debranching enzymes involved in amylopectin synthesis were upregulated at DAF7-DAF21 (Fig. 6). Taken together, we speculate that *OsCRLK2* may influence GT by altering the ratio of amylose to amylopectin as well as the fine structure of starch in rice grains.

### Haplotype Analysis of *OsCRLK2*

**Fig. 7:**
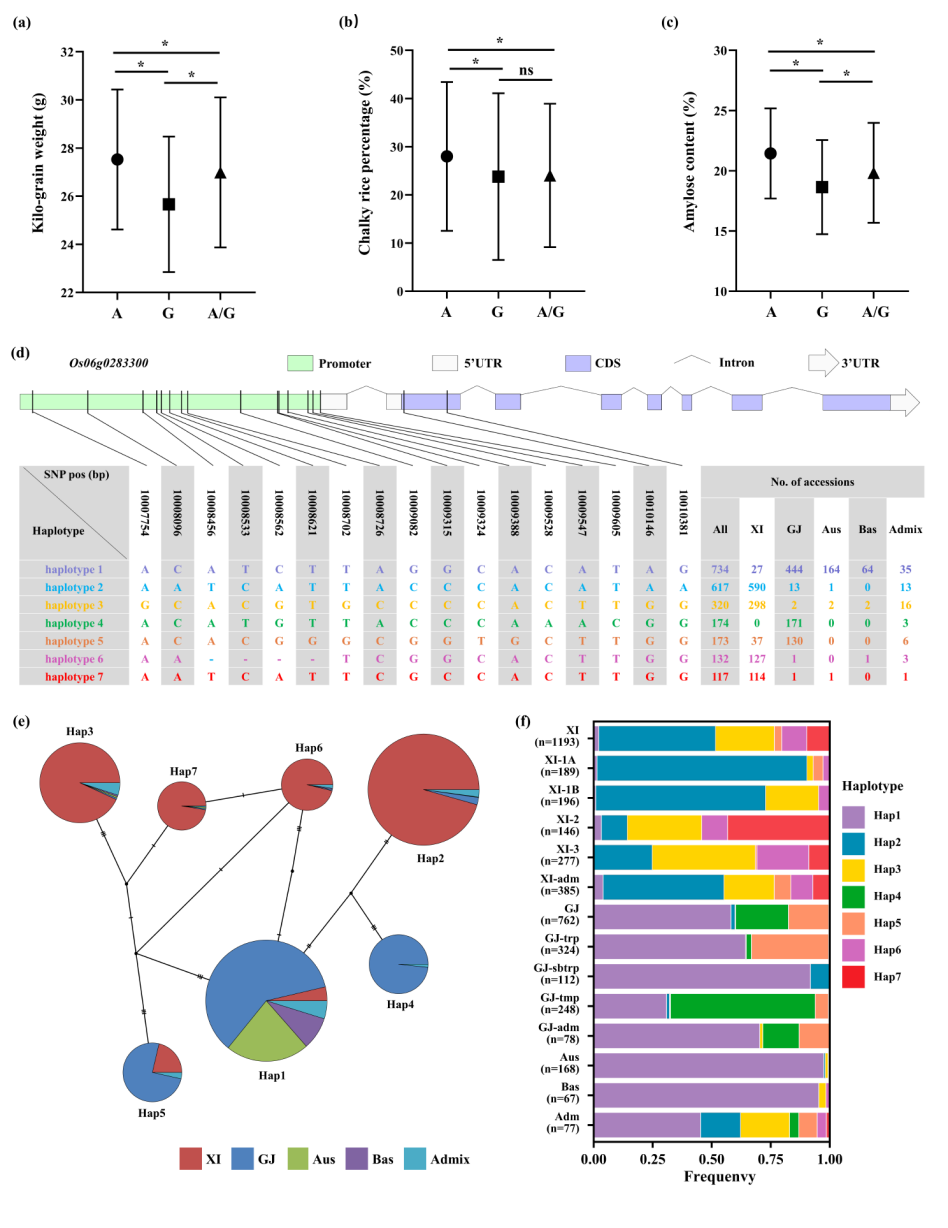
Genetic diversity of *OsCRLK2* in the RHRD and 3K RFGB datasets. Based on the RHRD dataset, the kilo-grain weight (**a**), chalky rice percentage (**b**), and amylose content (**c**) of *indica* hybrid rice were distinguished by key SNP within the *OsCRLK2* CDS. (**d**) Based on the 3K RFGB dataset, haplotypes of *OsCRLK2* were resolved using 15 SNPs in the promoter region and 2 SNPs in the CDS region (rare haplotypes represented by < 100 accessions are not shown). (**e**) Haplotype network of *OsCRLK2* in 3K RFGB. (**f**) Haplotype frequency of *OsCRLK2* in subpopulations of 3K RFGB

Using sequencing data from 349 natural population materials combined with RVA profiles for analysis, the results revealed that the 349 materials could be classified into three haplotypes based on *OsCRLK2*^*13*^ and *OsCRLK2*^*249*^. Among them, PKV, TV, and FV of Hap1 were significantly higher compared to those of Hap2 and Hap3, while the GT of Hap1 was significantly elevated compared to Hap2 (Fig.S4, Table S9). The RHRD database (http://ricehybridresource.cemps.ac.cn/#/) was utilized to assess kilo-grain weight, chalky rice percentage, and amylose content in *indica* hybrid rice. The key SNPs in the *OsCRLK2* CDS region can categorize *indica* hybrid rice into three distinct haplotypes: *OsCRLK2*^*13A*^, *OsCRLK2*^*13G*^, and *OsCRLK2*^*13A/G*^. The *OsCRLK2*^*13A*^ haplotype results in increased kilo-grain weight, chalky rice percentage, and amylose content. Conversely, the *OsCRLK2*^*13G*^ haplotype results in decreased kilo-grain weight, chalky rice percentage, and amylose content. In the case of the heterozygous site, the kilo-grain weight and amylose are intermediate (Fig. 7a–c). These results further substantiate the involvement of *OsCRLK2* in regulating rice quality.

Additionally, to analyze the evolutionary relationships of *OsCRLK2* homologs among rice subspecies, we examined *OsCRLK2* polymorphism in the 3K RFGB database (https://www.rmbreeding.cn/index.php). Fifteen SNPs in the promoter region and two SNPs in the CDS region revealed seven major *OsCRLK2* haplotypes (rare haplotypes occurring in < 100 accessions are not shown). The frequencies of these major haplotypes varied significantly between the *Xian-* and *Geng-*type cultivars. Specifically, Hap2, Hap3, Hap6, and Hap7 were predominantly of the *Xian-*type, whereas Hap1, Hap4, and Hap5 were primarily of the *Geng-*type (Fig. 7d–f). These findings imply substantial differentiation between *Xian* and *Geng* in *OsCRLK2*.

## Discussion

Rice quality collectively comprises appearance, texture, aroma, flavor, and nutritional composition. Rice exhibiting exceptional qualities is more favored by consumers and more competitive in the market (Zhao et al. 2022). Because of this, the development of exceptionally high-quality rice is a significant breeding objective. A comprehensive understanding of the regulatory network governing rice quality is necessary for breeders to make targeted selections and improvements in rice quality.

To that end, significant progress has been made in cloning genes associated with rice quality, and some of these genes have been applied in rice quality improvement breeding. For example, Jin et al. (Jin et al. 2010) used molecular marker-assisted selection to target the *Wx*, *SSIIa*, and *fgr* genes in ‘II-32B’ rice, resulting in the development of an improved ‘II-32B’ strain with enhanced aromatic properties, low amylose content, and a lower GT. Ni et al. (Ni et al. 2011) utilized molecular marker-assisted selection to replace *Wx*^*a*^ with *Wx*^*b*^ in the high-yielding cultivar ‘Xieyou57’, leading to improving eating and cooking quality (ECQ). Wang et al. (Wang et al. 2015b) introduced *GL7* and *gs3* into the sterile *indica* line ‘Yuefeng’, significantly increasing yield and improving the appearance of the rice grains. Wang et al. (Wang et al. 2015a) developed the high-quality, high-yielding hybrid *indica* cultivars ‘Taifengyou55’ and ‘Taifengyou208’ by aggregating *gs3* and *GW7*^*TFA*^. Zhou et al. (Zhou et al. 2019) employed CRISPR/Cas9 to edit *GS3*, *GW2*, and *Gn1a*, leading to increased grain length, grain width, and spikelet number in the triple mutants. Zeng et al. (Zeng et al. 2020) used CRISPR/Cas9 to edit the 5’ untranslated region (5’UTR) of *Wx* in ‘TaiFengB’ rice, resulting in significantly reduced amylose content without affecting yield. In fact, the quality of ‘TaiFengB’ rice was improved to such an extent that the grains resembled those of the premium *indica* rice ‘Huang Huazhan’ (HHZ) (Zeng et al. 2020). Huang et al. (Huang et al. 2021) utilized CRISPR/Cas9 to edit the starch biosynthesis genes *Wx* and *SSIIa*, resulting in improved ECQ. Hui et al. (Hui et al. 2022) edited *OsBADH2*, which affects aromatic quality, in the *indica* rice ‘HHZ’. Furthermore, through hybridization with the cytoplasmic male sterility (CMS) line ‘Taonong 1A’ (TN1A), they obtained a highly-fragrant three-line hybrid rice ‘B-Tao-You-Xiangzhan’ (BTYXZ) with improved viscosity compared to the WT ‘Tao-You-Xiangzhan’ (TYXZ) (Hui et al. 2022). Finally, Tan et al. (Tan et al. 2023) utilized CRISPR/Cas9 to edit regulatory elements in the promotor of *SLG7* in slender grain-type rice, ultimately increasing *SLG7* expression and producing a novel allelic variation associated with increased grain length:width ratio and reduced chalkiness. Such advancements have accelerated the breeding process, emphasizing the critical importance of comprehensively characterizing genes affecting rice quality.

These genes have evidently contributed to improving rice quality. However, given the complexity of the genetic and regulatory networks governing rice quality, many genes influencing rice quality remain unreported. Starch, being the primary component of rice, remains a central focus of research. Numerous studies have shown a significant correlation between RVA profiles and amylose content (Chen et al. 2021), which is consistent with our research findings (Table S3). RVA profiles exhibit considerable variability among materials with differing amylose contents, effectively reflecting their gelatinization and viscosity properties. Additionally, Tao et al. (Tao et al. 2019) have demonstrated that the distribution of amylose chain lengths and the overall size of amylopectin also impact RVA profiles.

In our study, two GT-associated QTLs (*qGT6.3* and *qGT6.4*) were identified by analyzing RVA profiles obtained in three environments. The candidate gene underlying *qGT6.3* was found to be *ALK*, which encodes soluble starch synthase IIa (SSIIa). Previous studies report that *ALK* has at least three haplotypes, in which *ALK*^*a*^ (A-GC) and *ALK*^*b*^ (G-TT) are associated with low GT and *ALK*^*C*^ (G-GC) is associated with high GT (Waters et al. 2005; Bao et al. 2006a; Tian et al. 2009; Gao et al. 2011). Only one differentially expressed gene (DEG) (*Os06g0283300*) was identified in the *qGT6.4* region. The HMMER web server (https://www.ebi.ac.uk/Tools/hmmer/) predicted that the protein encoded by *Os06g0283300* contains a transmembrane domain and an intracellular juxtamembrane domain, and may be an RLK (Liang and Zhou 2018).

Receptor-like kinases (RLKs) play important roles in plant growth, development, and signal transduction. For example, mutation of *ZmCR4* in maize or silencing of *OsCR4* in rice both result in the partial loss of the endospermic aleurone cell layer (Becraft PW et al. 1996; Pu et al. 2012). In *Arabidopsis*, AtVRLK1 signaling coordinates cell elongation and cell wall thickening during growth and development (Huang et al. 2018). Also in *Arabidopsis*, LecRK-VIII.2 influences silique number, seed size, and seed number to determine seed yield (Xiao et al. 2021). *MIS2* controls grain size through coordinated regulation of epidermal cell size and cell number (Chun et al. 2020). However, little is known about how RLKs affect rice quality.

The *OsCRLK2* homolog *AtCRLK2* in *Arabidopsis* appears to be upstream of the mitogen-activated protein kinase (MAPK) cascade, MPK3 and MPK6 phosphorylate ICE1, promoting its degradation and thereby negatively regulating the cold response, while AtCRLK2/AtCRLK1 suppress the cold activation of MPK3/6, thus modulating the plant cold response (Zhao et al. 2017; Liu and Zhou 2018). In rice, mutations in *oscrlk2* result in decreased GT, AAC, and viscosity, as well as increased chalkiness (Fig. 5e–j). We speculate that *oscrlk2* may influence protein translational modifications (PTMs) of rice quality-related genes through the MAPK cascade, thereby affecting rice quality (Li et al. 2019; Guo et al. 2020).

In addition, we conducted qRT‒PCR analysis of starch biosynthesis-related and sucrose synthasegenes during the grain filling stage (DAF7-DAF28) in both WT and mutant plants. Compared to WT, the expression levels of most starch biosynthesis-related genes showed a downward trend during the grain filling stage, particularly the major functional gene for starch biosynthesis, *Wx*, which exhibited significantly reduced expression levels in the mutant from DAF7 to DAF28. Meanwhile, the expression levels of genes encoding soluble starch synthase, branching enzymes and debranching enzymes involved in amylopectin synthesis were also alteredduring the DAF7 to DAF21 period in mutant. Branching enzymes introduce new branches, while debranching enzymes remove incorrect branching points, thereby facilitating the formation of a crystallizable helical structure in amylopectin (Tian et al. 2009). These alterations may lead to changes in the branching ratio of amylopectin and the fine structure of starch in mutants. Interestingly, we observed a significant upregulation in the expression of most sucrose synthase genes in mutants. Sucrose synthase mainly participates in sucrose metabolism in tissues where sucrose is consumed, but its metabolic pathways are reversible within plant tissues. The sucrose content was significantly higher in *oscrlk2* compared to the WT (Fig. 5i)., speculating that the change in sucrose content in *oscrlk2* may be attributed to indirect effects resulting from reduced accumulation of amylose and altered expression of starch synthesis-related genes, and some sucrose synthase in *oscrlk2* may potentially catalyze sucrose synthesis.

Notably, haplotype analysis using natural population materials and the RHRD and 3 K RFGB databases revealed significant differentiation of *OsCRLK2* between *indica* and *japonica* rice (Wang et al. 2019; Gu et al. 2023). Among the 349 natural population materials, Hap1 significantly increased the PKV, TV, FV, and GT of starch. In *indica* hybrid rice, *OsCRLK2*^*13A*^ increases kilo-grain weight, chalky rice percentage, and amylose content, while *OsCRLK2*^*13G*^ decreases kilo-grain weight, chalky rice percentage, and amylose content. Furthermore, *OsCRLK2*^*13A/G*^ results in intermediate kilo-grain weight and amylose content (Gu et al. 2023). Overall, the results of this study provide a novel genetic resource which can be utilized for advancing research into improved rice quality.

### Electronic Supplementary Material

Below is the link to the electronic supplementary material.

Supplementary Material 1

Supplementary Material 2

Supplementary Material 3

Supplementary Material 4

Supplementary Material 5

## Data Availability

Annotated function of differentially expressed genes identified between PYZX and P02428 are available within the supplementary information files. The genotypes and phenotypes for approximately 6000 rice accessions data that support the findings of this study are available from RFGB (https://www.rmbreeding.cn/index.php) and RHRD (http://ricehybridresource.cemps.ac.cn/#/). Rice seeds are available from the National Engineering Research Center of Plant Space Breeding, PR China.

## Abbreviations

RIL: recombinant inbred lines
QTL: quantitative trait loci
LOD: limit of detection
RVA: Rapid Visco Analyze
GC: gel consistency
GT: gelatinization temperature
PKV: Peak viscosity
TV: Trough viscosity
BDV: Breakdown value
FV: Final viscosity
SBV: Setback value
CSV: consistency viscosity
PKT: Peak Time
AAC: Apparent amylose content
