# Supplementary figures and images for: OsCRLK2, a Receptor-Like Kinase Identified by QTL Analysis, is Involved in the Regulation of Rice Quality
Source: Rice (N Y). 2024 Apr 8;17:24. doi: 10.1186/s12284-024-00702-2 (PMC11001810; doi:10.1186/s12284-024-00702-2)

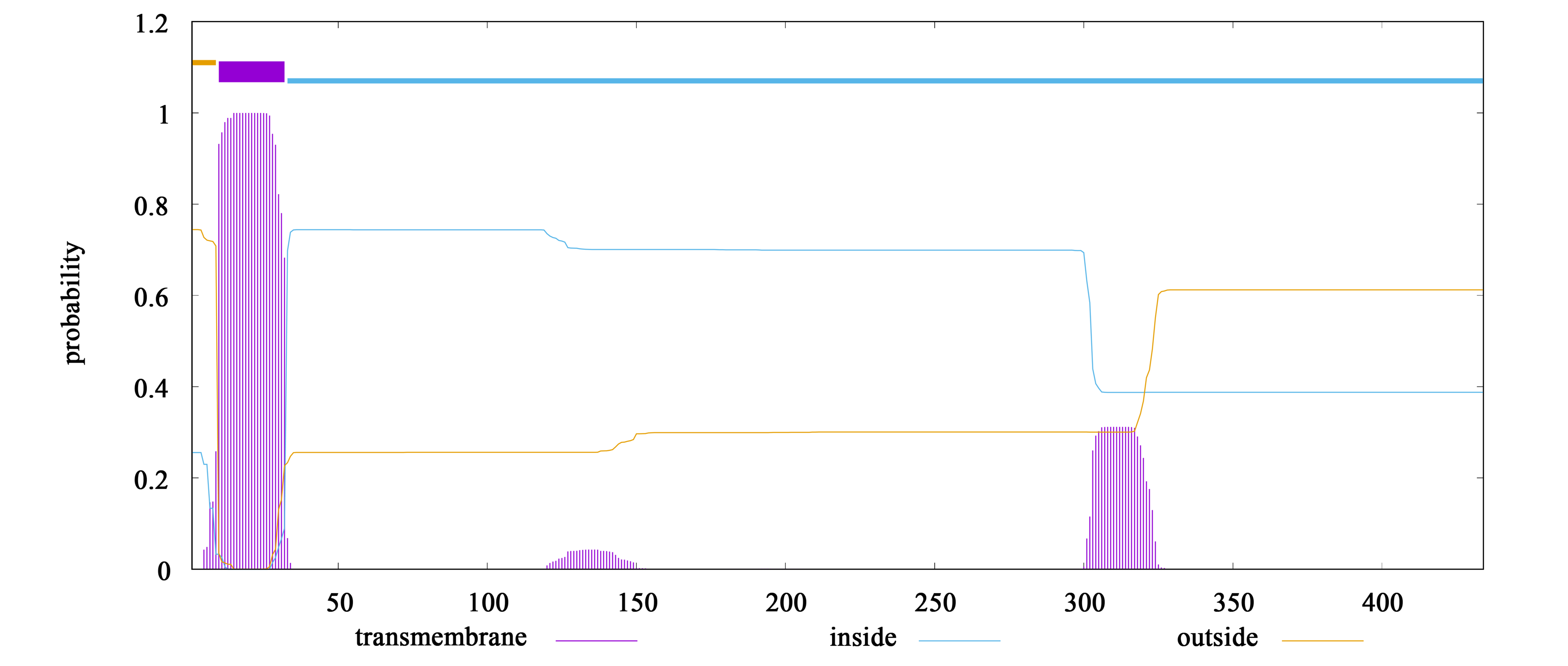

Supplement: Supplementary file 2 — Supplementary Material 2 [file 12284_2024_702_MOESM2_ESM.tif]

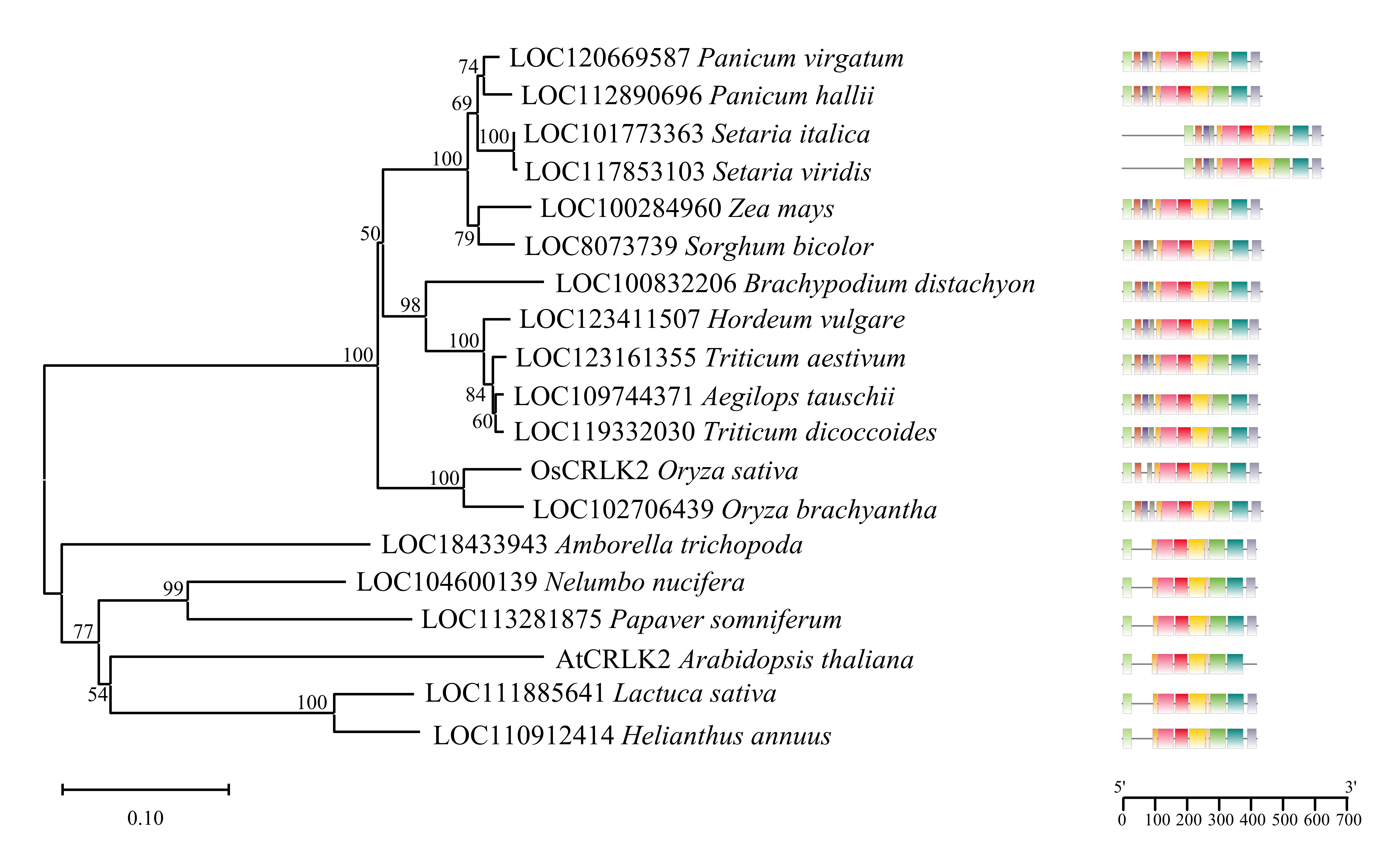

Supplement: Supplementary file 3 — Supplementary Material 3 [file 12284_2024_702_MOESM3_ESM.tif]

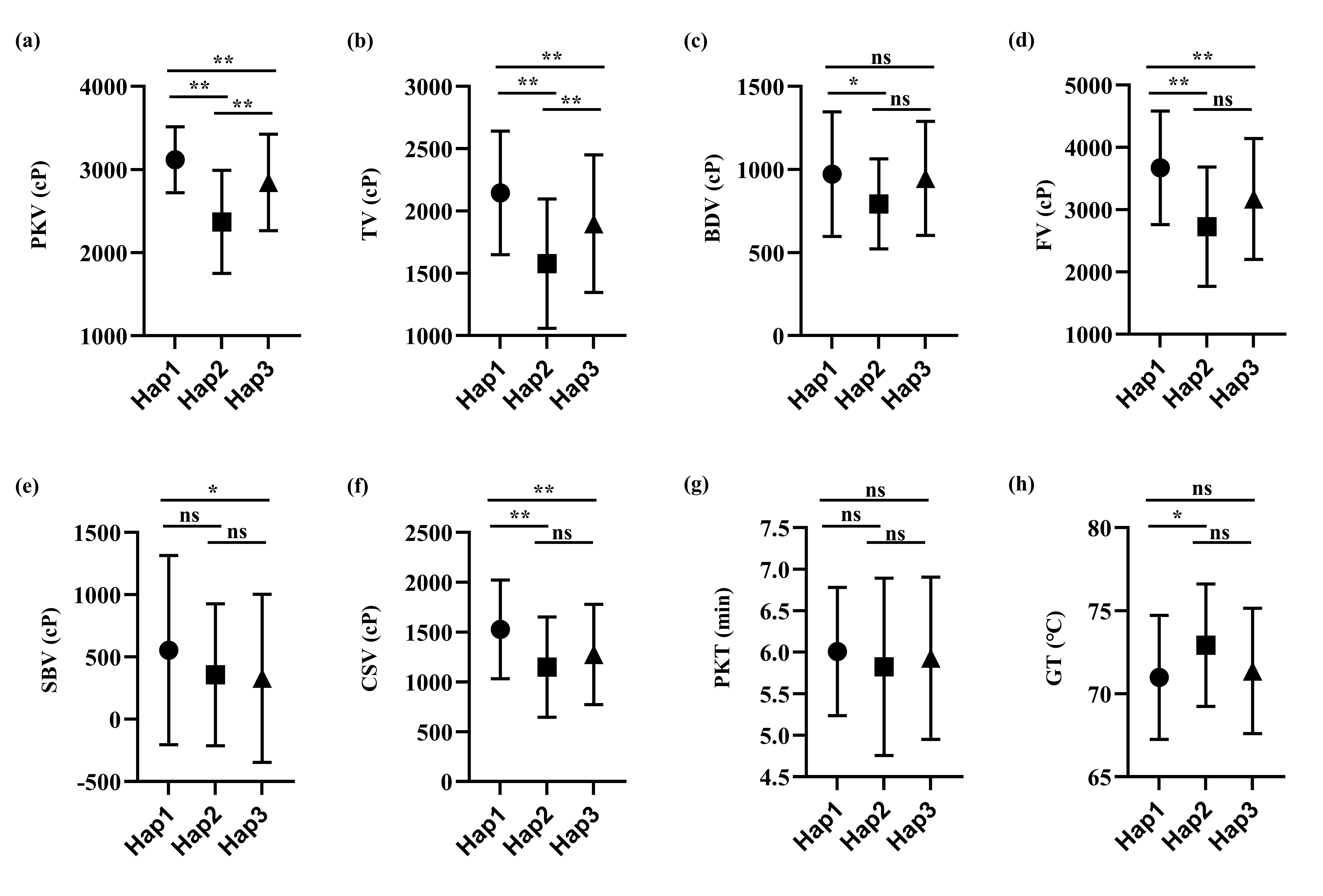

Supplement: Supplementary file 4 — Supplementary Material 4 [file 12284_2024_702_MOESM4_ESM.tif]
